# Glutathione reductase modulates endogenous oxidative stress and affects growth and virulence in *Avibacterium paragallinarum*

**DOI:** 10.1186/s13567-024-01388-6

**Published:** 2025-01-02

**Authors:** Yan Zhi, Chen Mei, Zhenyi Liu, Ying Liu, Hongjun Wang

**Affiliations:** Institute of Animal Husbandry and Veterinary Medicine, Beijing Academy of Agriculture and Forestry Sciences, Beijing, China

**Keywords:** Glutathione reductase, *Av. paragallinarum*, Oxidative stress, Bacterial virulence, Redox homeostasis, Metabolic adaptation

## Abstract

Glutathione reductase (GR) plays a pivotal role in managing oxidative stress, a process crucial for microbial virulence and adaptation, yet it has not been extensively explored in bacteria such as *Avibacterium paragallinarum* (*Av. paragallinarum*). This study examined the specific roles of GR in *Av. paragallinarum*, focusing on how GR modulates the bacterium’s response to oxidative stress and impacts its pathogenic behavior. Using gene knockouts together with transcriptomic and metabolomic profiling, we identified an important shift in redox balance due to GR deficiency, which disrupted energy metabolism and weakened the oxidative stress defense, culminating in a notable decline in virulence. In addition, decreased growth rates, reduced biofilm production, and weakened macrophage interactions were observed in GR-deficient strains. Notably, our findings reveal a sophisticated adaptation mechanism wherein the bacterium recalibrated its metabolic pathways in response to GR deficiency without fully restoring virulence. Our in vivo studies further highlight the pivotal role of GR in pathogen fitness. Together, our findings connect GR-mediated redox control to bacterial virulence, thereby furthering the understanding of microbial adaptation and positioning GR as a potential antimicrobial target. Our insights into the GR-centric regulatory network pave the way for leveraging bacterial redox mechanisms in the development of novel antimicrobial therapies, highlighting the importance of oxidative stress management in bacterial pathogenicity.

**Supplementary Information:**

The online version contains supplementary material available at 10.1186/s13567-024-01388-6.

## Introduction

*Avibacterium paragallinarum* (*Av. paragallinarum*), the causative agent of infectious coryza in chickens, is known for causing severe upper respiratory tract inflammation, as well as significantly decreasing egg production and growth in laying hens [1]. Despite its reclassification from *Haemophilus* to *Avibacterium* genus [2], due to a lack of nicotinamide adenine dinucleotide (NAD) biosynthetic capabilities, the vast majority of *Av. paragallinarum* strains are strictly dependent on NAD for growth [3]. The dynamic redox interplay between NAD + and its reduced counterpart NADH is fundamental to metabolic processes, particularly in maintaining cellular energy balance and redox homeostasis [4, 5], affecting cellular energy balance and stress response mechanisms [6]. However, the specific regulatory roles and pathways of NAD utilization in NAD-dependent bacteria like *Av. paragallinarum* remain poorly characterized, highlighting a significant gap in our understanding of bacterial metabolic and physiological intricacies.

The redox balance between NAD + /NADH and the glutathione system (GSH/GSSG) is pivotal for cellular stability and oxidative stress management [7–9]. Central to this balance is glutathione reductase (GR), which uses nicotinamide adenine dinucleotide phosphate (NADPH) to facilitate the reduction of glutathione disulfide (GSSG) to glutathione (GSH), thus equipping the cell to counteract oxidative stress [10]. The generation of NADPH, mainly through the pentose phosphate pathway, is intricately linked to the metabolic state of NAD + /NADH, underscoring the interdependence of these redox couples in maintaining cellular health [11, 12].

The important roles of NAD + /NADH and GSH/GSSG ratios in bacterial physiology have been documented. For example, in *Streptococcus pneumoniae*, significant shifts in the NAD + /NADH ratio impact the bacterial energy pool and ATP concentration, subsequently increasing the susceptibility of Gram-positive bacteria to antibiotics [13]. Similarly, in *Streptococcus* mutants, the *GshT* gene affects GSH capture by modulating GSSG levels [14]. However, their implications for the pathogenicity and virulence of bacteria such as *Av. paragallinarum* remain underexplored. The adaptive response of these pathogens to endogenous oxidative stress, as distinct from exogenous stressors, is a key area that requires further elucidation. Given the protective role of GR across species – from plant stress resilience [15], through lung development and hyperoxia response in neonatal mice [16], to defense mechanisms against gram-negative bacterial infections in sepsis models [17]—this enzyme emerges as an important research focus. Thus, the current study focuses on GR, specifically aiming to elucidate its complex role in *Av. paragallinarum* to further our understanding of the bacterial oxidative stress response.

In this study, we aimed to elucidate the physiological roles of GR within the GSH cycle in *Av. paragallinarum*. By utilized gene knockout techniques, we sought to investigate how GR deficiency affects the bacterium’s metabolism and virulence. Understanding these mechanisms could provide insights into the broader context of bacterial oxidative stress responses and potentially identify new targets for antimicrobial therapy.

## Materials and methods

### Ethics approval

All procedures involving animals were conducted in strict accordance with the International Guiding Principles for Biomedical Research Involving Animals, ensuring the highest standards of animal welfare (NIH Guiding Principles, 2012). This study received ethical approval from the Institute of Animal Husbandry and Veterinary Medicine’s Animal Care and Use Committee (Permit number: IHVM11-2302–5). Efforts to minimize animal suffering and promote welfare were paramount, and included the incorporation of anesthetics, refined handling techniques, and rigorous application of the three Rs principle (replacement, reduction, and refinement) to lessen the impact on participating animals [18].

### Bacterial strains, plasmids, and growth conditions

*Av. paragallinarum* was propagated in Tryptic Soy Agar (TSA) and Tryptic Soy Broth (TSB) (Becton Dickinson, USA) enriched with 10% chicken serum and 25 μg/mL NAD, and incubated at 37 °C for 16 h. Satellite growth assessments were carried out in 5% sheep blood agar (Qingdao Hope Bio-Technology Co., Ltd., China). Incubations were performed at 37 °C with agar plates placed in an atmosphere of 5% CO_2_ and broth cultures aerobically shaken at 170 rpm. Details on strains, vectors, and primers are provided in Additional files 1–3. For *Escherichia coli* (*E. coli*) cultures, Luria Bertani (LB) broth supplemented with 50 mg/mL kanamycin was used as required.

### Bacterial growth rate determination

To determine the growth rate of *Av. paragallinarum*, several representative colonies were selected from a supplemented TSA plate and cultured in supplemented TSB for 16 h. The initial bacterial count was standardized to 1 × 10^8^ CFU/mL by adjusting the OD_600_ of the cultures. For the wild-type (WT) strain, an OD_600_ of 0.35 corresponded to 1 × 10^8^ CFU/mL, while for the GR knockout (ΔGR) strain, an OD_600_ of 0.60 was required to achieve the same viable count. A 2% inoculum (2 mL in a 100 mL broth culture) of this culture was then transferred into 100 mL TSB and incubated for 24 h at 37 °C. Growth monitoring involved aseptic sampling from the culture broth at predetermined intervals (initially at 2 and 4 h, then every 30 min from 4 to 9 h, and every 2 h from 9 to 24 h), followed by serial tenfold dilutions and plating on supplemented TSA. Viable counts were determined after 24 h, using the formula: CFU/mL = number of colonies × 10 × dilution factor.

### Construction of mutants

**Figure 1:**
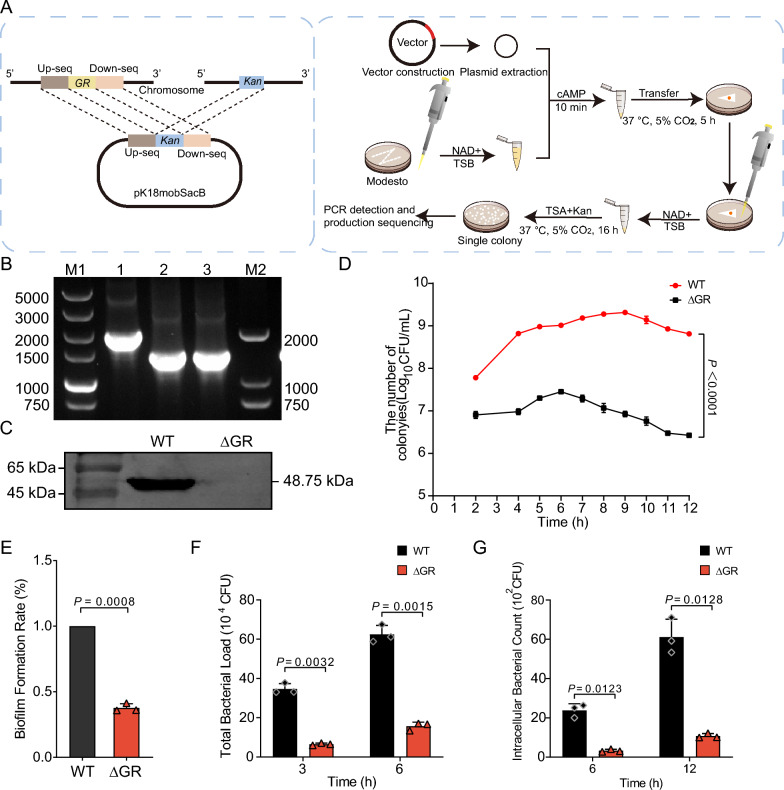
**GR knockout in *Av. paragallinarum* affects growth and host–pathogen interactions. A** Schematic diagram showing the construction strategy for the GR knockout mutant in Av. paragallinarum using a kanamycin resistance cassette (Kan). The schematic outlines the homologous recombination process used to replace the GR gene with the Kan cassette, detailing the steps for successful gene transfer and integration into the bacterial chromosome. **B** PCR analysis and product sequencing confirmed the knockout of the GR gene. Lane 1 represents the wild-type strain (WT, 1798 bp), lane 2 represents the positive control with the plasmid containing the kanamycin resistance gene (1258 bp), and lane 3 represents the GR knockout strain (ΔGR, 1258 bp). **C** Western blot analysis was used to show the absence of GR protein in the GR mutant (ΔGR) strain compared to the wild type (WT) strain. The target protein size is approximately 48.75 kDa. The absence of a band in the ΔGR lane indicates successful GR gene knockout, while the presence of a band in the WT lane confirms the expression of the GR protein. **D** Growth curve comparison of WT and ΔGR strains under aerobic conditions. **E** Quantitative assessment of biofilm formation in WT and ΔGR strains. **F** The macrophage interaction assay was used to assess the adhesion capability of WT and ΔGR strains over time. **G** The invasion assay was used to evaluate the invasion capabilities of WT and ΔGR strains into HD11 chicken macrophages.

Natural transformation was used to construct *Av. paragallinarum* mutant strains as described previously [19]. Specifically, homologous arms of the GR gene, which is located between base pairs 801,093 and 802,448 in the *Av. paragallinarum* genome, were amplified, linked to a kanamycin resistance gene, and inserted into the PK18mobsacB plasmid. This construct was then introduced into *E. coli* DH5-α for plasmid extraction. The recipient strain, Modesto, was activated on TSA plates and incubated overnight at 37 °C. Then, the strains were resuspended in TSB to adjust the OD_600_ to 0.9–1.0, which was determined through multiple trials to be the most effective concentration for successful plasmid uptake during transformation in *Av. paragallinarum*. Next, 20 μL cAMP and 10 μL plasmid (around 1 μg) were sequentially added to this suspension, each followed by a 10-min incubation. A negative control using 10 μL sterile water was also prepared. The mix was then plated on TSA and incubated for 5 h at 37 °C. After washing with TSB, the bacteria were plated on TSA containing kanamycin (50 µg/mL) and incubated at 37 °C. to select transformants. PCR was used to confirm that the gene had been deleted in the resulting transformants, as shown in Figure 1A. The specific primers were designed to target the regions flanking the GR gene insertion site, ensuring the accurate identification of successful knockouts.

### Extracellular metabolite analysis

Redox state dynamics were assessed in *Av. paragallinarum* by quantifying key redox metabolites in wild-type (WT) and GR mutant strains using kits from Beijing Biocloud Technology Co., Ltd. Cellular redox homeostasis was assessed by measuring (i) reduced and oxidized glutathione (GSH and GSSG) levels; (ii) total, selenium-containing, and non-selenium glutathione peroxidase (GPx) levels; and (iii) NAD + /NADH and NADP + /NADPH ratios. In addition, total reactive oxygen species (ROS) levels were measured in the culture supernatant, intracellular contents (released after bacterial cell lysis), and growth medium using a ROS Assay Kit (Genmed Scientifics Inc., USA, Catalog No. GMS10016.14 v.A), designed for bacterial systems. Colonies from TSA plates were transferred to 100 mL of TSB medium and incubated at 37 °C until reaching a concentration of approximately 1 × 10^8^ CFU/mL. Subsequently, a 2% (v/v) inoculum (2 mL) was used to inoculate a fresh 100 mL TSB broth, which was then incubated for an additional 5 h at 37 °C to reach the logarithmic phase. The cultures were subsequently normalized to a concentration of 1 × 10^8^ CFU/mL for subsequent experiments, ensuring representation of peak metabolic activity under standard conditions.

### Biofilm formation assay

Biofilm formation by *Av. paragallinarum* was assessed using a previously described method [20] with minor modifications. Briefly, bacterial suspensions were adjusted to 1 × 10^6^ CFU/mL, and then further diluted 1:50 in TSB. A 200 μL aliquot of this dilution was dispensed into each well of a 96-well plate, with TSB serving as the blank control. Plates were incubated at 37 °C for 48 h. Post-incubation, wells were washed three times with PBS, and excess liquid was removed by inverting the plate on absorbent paper. Each well was fixed with 150 μL of 99% methanol for 20 min, air-dried, and then stained with 1% crystal violet for 30 min. After washing with PBS until clear, the wells were air-dried, and 150 μL 33% acetic acid was added to solubilize the crystal violet. A microplate reader was used to measure the absorbance at OD_570_. The assay was replicated in triplicate and used to quantify biofilm formation in both WT and mutant strains.

### Macrophage internalization assay

The effects of GR deletion on the ability of *Av. paragallinarum* to adhere to and invade the HD11 chicken macrophage cell line were evaluated, as described previously [21]. Briefly, HD11 cells were cultured in 12-well plates and exposed to 1 × 10^8^ CFU of WT and GR mutant strains.

For the internalization assay, cells were incubated with bacteria for 3 and 6 h at 37 °C, after which the plates were washed three times with PBS to remove non-adherent bacteria. The remaining internalized bacteria were quantified by lysing HD11 cells with 1% Triton X-100 in PBS, followed by dilution plating for bacterial counts.

To distinguish between surface-adhered and internalized bacteria, following the initial incubation and washing steps, HD11 cells were treated with 50 μg/mL cefalexin for 2 h to kill extracellular bacteria. Next, cells were washed with PBS, incubated for a further 6 to 12 h, lysed, and bacterial enumeration was conducted to assess the internalization capabilities.

### Animal virulence testing

The effects of GR deletion on the virulence of *Av. paragallinarum* were examined in 42-day-old SPF chickens (*n* = 105) obtained from Beijing Vital River Laboratory Animal Technology Co., Ltd. Chickens were divided into seven groups (*n* = 5 chickens/group) (WT infection, five GR knockout dosage levels, and control), and acclimated for a week before experimentation. Chickens were housed in individual SPF units and had free access to feed and water throughout the experiment.

**Figure 2:**
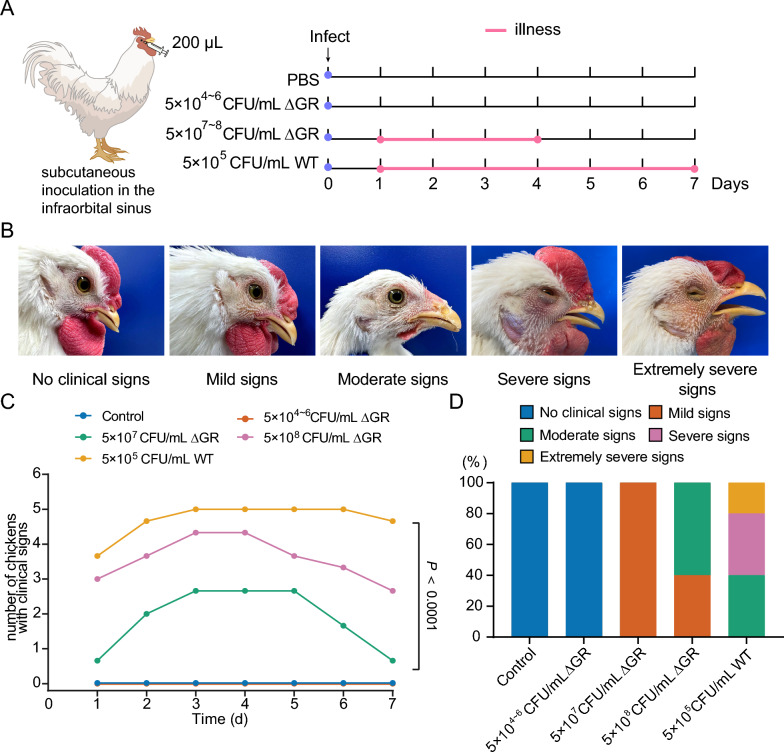
**In vivo pathogenicity studies reveal diminished virulence in GR-knockout** ***Av. paragallinarum*** **. A**Schematic showing the design of the animal virulence assay. Chickens were subcutaneously inoculated with different doses of *Av. paragallinarum* strains in the infraorbital sinus and monitored for 7 days. **B** Disease severity in chickens infected with different doses of the ΔGR strain compared to the WT strain. **C** Disease incidence in chickens exposed to different doses of the GR mutant (ΔGR) strain compared to the wild-type (WT) strain (*P* < 0.0001). **D** The disease severity categorization model was used to determine virulence levels.

Infection doses were based on our preliminary findings. The WT group received a subcutaneous inoculation of 5 × 10^5^ CFU in the infraorbital sinus, GR-knockout group received 5 × 10^4^ to 5 × 10^8^ CFU, and controls were administered PBS. Daily monitoring, which lasted for 7 days, included assessments of alertness, body weight, feed intake, and mobility [22]. Similar to previously reported studies [23], the severity of nasal discharge and facial swelling in the infected chickens was scored on the following scale: 0 for no clinical symptoms; 1 for mild signs (slight facial swelling); 2 for moderate signs (moderate facial swelling and nasal discharge); 3 for severe signs (severe facial swelling, nasal discharge, tearing, half-closed eyes); and 4 for extremely severe signs (extremely severe facial swelling, copious nasal discharge, tearing, fully closed eyes). The design of our animal virulence assay is shown in Figure 2A. The scoring model, which categorizes disease severity levels, is shown in Figure 2B. To ensure the rigor of the experiment, these tests were repeated three times at different intervals.

### Transcriptome sequencing

Using the Illumina next-generation sequencing platform with a PE150 sequencing strategy, we performed transcriptomic sequencing on both WT and GR mutant strains of *Av. paragallinarum*. Transcriptome sequencing was carried out by first activating the strains and then inoculating them into TSB medium enriched with 10% chicken serum and 0.0025% NAD, with the cultures standardized to a density of 1 × 10^8^ CFU/mL. Cultures were incubated for 5 h at 37 °C with shaking, and then harvested in log phase. The bacterial cultures were then normalized by adjusting the OD_600_ to ensure that the starting cell density was consistent across all samples. This normalization was validated by actual colony counts to confirm that each sample contained 1 × 10^8^ CFU/mL before RNA extraction. Bacteria were washed three times with PBS and centrifuged to remove NAD from the culture medium. Samples were subsequently prepared for transcriptomic sequencing, with each experimental condition replicated three times. Sequencing was performed by Allwegene Technology Co., Ltd (Beijing, China).

### LC–MS/MS analysis of energy metabolites

LC–MS/MS was used to analyze the intracellular metabolites in WT and GR mutant strains of *Av. paragallinarum* during the same growth phase. Samples were collected as described in sub-section “Transcriptome sequencing”, above, ensuring consistency and accuracy across experiments. Similar to the transcriptomic analysis, the bacterial cultures were normalized to 1 × 10^8^ CFU/mL after reaching the mid-log phase. This normalization step ensured that the metabolite concentrations reflected the true physiological differences between the WT and ΔGR strains, rather than differences in growth rates. The samples were then processed for metabolomic analysis, which was conducted by Allwegene Technology Co., Ltd (Beijing, China).

### Whole-genome sequencing

To validate the genomic integrity of the *Av. paragallinarum* standard strain Modesto, we performed comprehensive resequencing of its entire genome. The newly generated sequence data have been submitted to the NCBI GenBank, accessible under accession number CP095161. For clarity and to differentiate from previously available Modesto sequences, this strain has been designated as “M”. This sequencing effort was undertaken in collaboration with the Beijing Genomics Institute (BGI).

### Validation of differentially expressed genes (DEGs) by reverse transcription-quantitative PCR (RT-qPCR)

The DEGs identified in our transcriptomic studies were validated by RT-qPCR using the primer sequences shown in Additional file 3. RT-qPCR was carried out using the 2 × Hieff UNICON^®^ Universal Blue qPCR Master Mix (Yeasen, Shanghai, China) under the following amplification conditions: Uracil-DNA Glycosylase (UDG) activation at 50 °C for 2 min, initial denaturation at 95 °C for 2 min, then 40 cycles of 95 °C for 15 s and 60 °C for 30 s for denaturation and annealing/extension, respectively, concluding with a melting curve analysis. Experiments were carried out in triplicate. The 16 s rRNA gene served as the internal control, and gene expression fold changes were calculated using the 2^−ΔΔCT^ method, with PCR efficiency confirmed to be consistent across all targets [24]. Data were analyzed with GraphPad Prism 8 [25].

### Western blotting analysis

Western blot analysis was performed to confirm the absence of GR protein expression in our constructed GR mutant strain of *Av. paragallinarum*. Protein extracts from both WT and GR mutant strains were obtained through ultrasonication, separated by 10% SDS-PAGE, and electroblotted onto membranes at 100 V for 90 min. Then, the membranes were blocked and incubated overnight at 4 °C with an anti-GR primary antibody of 1:1000 (EPR7238, ab124995, Abcam), followed by incubation with IRDye 800CW goat anti-rabbit IgG (H + L) secondary antibody (926–32,211, LI-COR) at a dilution of 1:15 000 for 1 h the next day. Protein bands were visualized using the Odyssey infrared imaging system (LI-COR Biosciences, Lincoln, NE, USA), and confirmed that GR protein expression was disrupted in the GR mutant strain.

### Statistical analysis

Statistical analysis was conducted using GraphPad Prism (Version 8.0, San Diego, USA), with results presented as the mean ± SD. Differences between two groups were evaluated using an unpaired Student’s *t*-test, and comparisons among multiple groups were performed with one-way ANOVA. For RT-qPCR, the relative quantification method (2^−ΔΔCT^) was utilized. For the animal virulence testing, data were analyzed using the non-parametric Mann–Whitney U test, which is more appropriate for small sample sizes and does not assume normal distribution of the data. A *P*-value < 0.05 was deemed statistically significant.

## Results

### Phenotypic analysis and macrophage interactions in GR-knockout *Av. paragallinarum*

We successfully generated a GR-knockout mutant of *Av. paragallinarum*. PCR confirmed that the GR gene (1356 bp) had been replaced with a kanamycin resistance cassette (816 bp), and western blot analyses confirmed that the GR protein was no longer expressed in the mutant strain (Figures 1B and C). The absence of the GR protein band in the ΔGR group, as seen in Figure 1C, is due to the successful knockout of the GR gene, resulting in no expression of the GR protein. Our phenotypic analysis revealed a notable decline in the peak growth rate of the GR mutant strain, highlighting the essential role of GR in sustaining bacterial proliferation (Figure 1D). These findings further suggest that GR has an important role in regulating the bacterial growth cycle and its resistance to oxidative stress. Strikingly, we observed a pronounced decrease in biofilm formation within the GR-deficient strain (Figure 1E). Given the pivotal role of biofilms in bacterial virulence and the establishment of persistent infections, these findings indicate a potential alteration in the pathogenic profile of the mutant strain, which may affect its ability to form protective communities against environmental stresses and antimicrobial agents. In comparison to the WT, assays evaluating interactions with HD11 chicken macrophages revealed a substantial impairment in the adhesion and invasion capabilities of the GR mutant strain (Figures 1F and G). This compromised interaction with host defense cells not only highlights the importance of GR in facilitating successful host–pathogen dynamics but also suggests a broader influence on the organism’s pathogenic mechanisms. The reduced interaction between the GR mutant strain and HD11 chicken macrophages further point to an intricate relationship between oxidative stress management and the virulence strategy of the bacterium, further verifying the important role of GR in mediating effective responses to the host immune system.

### Virulence assessment in *Av. paragallinarum* post-GR knockout: an in vivo study

Our in vitro findings showed diminished macrophage interaction and biofilm formation suggest that knockout of GR in *Av. paragallinarum* results in a potential decrease in virulence. Thus, we next conducted in vivo experiments within the natural host to conclusively determine changes in virulence. Our in vivo data revealed a significant reduction in virulence for the GR knockout strain compared to WT. Specifically, a dose of 5 × 10^5^ CFU/mL of the WT strain led to the rapid onset of illness in chickens within approximately 24 h, with the duration of the illness lasting for about 7 days. Conversely, illness was not induced in the GR mutant strain following administration of various doses ranging from 5 × 10^4^ CFU/mL to 5 × 10^6^ CFU/mL. Illness was only observed after administration of a higher dose of 5 × 10^7^ CFU/mL, with a significant increase in incidence at 5 × 10^8^ CFU/mL, which was still significantly lower than the WT group (*P* < 0.0001) (Figure 2C). Utilizing an established scoring model for infectious coryza, chickens infected with the GR mutant strain at doses of 5 × 10^7^ and 5 × 10^8^ CFU/mL exhibited less severe symptoms and a shorter duration of illness compared to the WT (Figure 2D). In summary, the absence of GR markedly attenuated the virulence of *Av. paragallinarum*, as substantiated by our in vivo assessments based primarily on descriptive analysis. These findings not only highlight the important role of GR in maintaining bacterial virulence but also demonstrate the complex interplay between metabolic regulation and pathogenicity in *Av. paragallinarum*, offering profound insights into how metabolic enzymes influence mechanisms of bacterial virulence.

### Transcriptomic alterations following GR knockout in *Av. paragallinarum*

**Figure 3:**
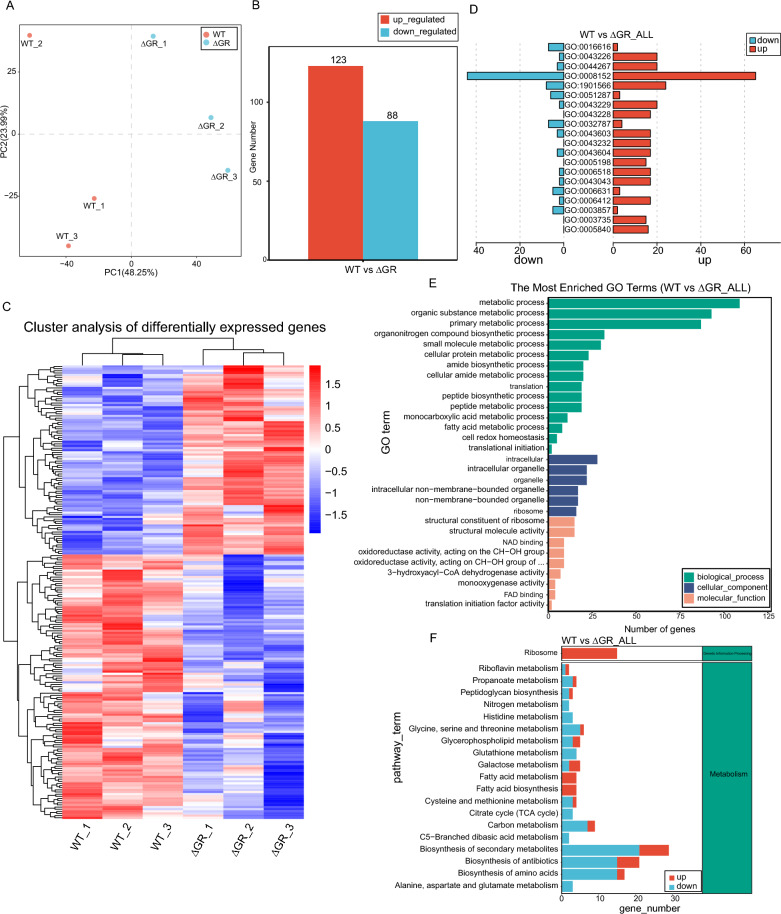
**Transcriptomic characteristics and functional annotation before and after GR knockout in *Av. paragallinarum*. A** PCA was used to compare the transcriptional profiles of wild-type (WT) and GR-knockout (ΔGR) strains. **B** Identification of 211 DEGs including123 upregulated and 88 downregulated genes. **C** Heatmap showing the cluster analysis of DEGs and the distinct expression profiles of upregulated and downregulated genes in ΔGR strains. **D** GO enrichment analysis of the DEGs shown as a bar graph. **E** Detailed GO enrichment analysis of the upregulated and downregulated DEGs. **F** Comparative analysis of metabolic pathways.

After quality control analysis (Additional file 4), the sequencing data were aligned and transcript assembly was performed with Bowtie2 and Rockhopper software using *Av. paragallinarum* ESV-135 (Genome assembly ASM1176560v1) as the reference genome. Principal component analysis (PCA) revealed distinct transcriptional profiles between the WT and GR mutant strains (Figure 3A). Closer examination revealed 211 DEGs when comparing the GR mutant strain to the WT strain, with 123 genes upregulated and 88 genes downregulated in the GR mutant strain (Figure 3B). Cluster analysis of these DEGs was visualized using a heatmap (Figure 3C).

Gene Ontology (GO) enrichment analysis, presented as circos and bar graphs (Figure 3D), indicated that metabolic processes were the most affected pathways. Upon further examination, upregulated genes were found to predominantly influence metabolic processes, particularly in organic substance metabolic processes and cellular nitrogen compound metabolic processes. In contrast, downregulated genes were mainly associated with small-molecule metabolic processes and regulation of cellular processes. Our differential enrichment data suggest a complex regulatory pattern, where upregulated genes may contribute to increased metabolic output, while downregulated genes reflect potential reductions in biosynthetic pathways and cellular regulatory mechanisms (Figure 3E). Kyoto Encyclopedia of Genes and Genomes (KEGG) pathway enrichment analysis revealed that the absence of GR primarily manifested as a gene regulation pattern against the backdrop of metabolic and genetic information processing pathways. Specifically, the ribosome pathway showed major gene upregulation, indicating enhanced translational activity. In contrast, pathways involved in metabolism, such as “alanine, aspartate, and glutamate metabolism”, “biosynthesis of secondary metabolites”, and “C5-branched dibasic acid metabolism”, displayed a balance of upregulated and downregulated genes. These findings suggested subtle regulatory adjustments within these pathways, potentially reflecting a complex biochemical response to the cellular state in the study (Figure 3F). Further details and supplementary analyses can be found in Additional file 5.

### Confirmation of DEGs in GR-knockout *Av. paragallinarum*

**Figure 4:**
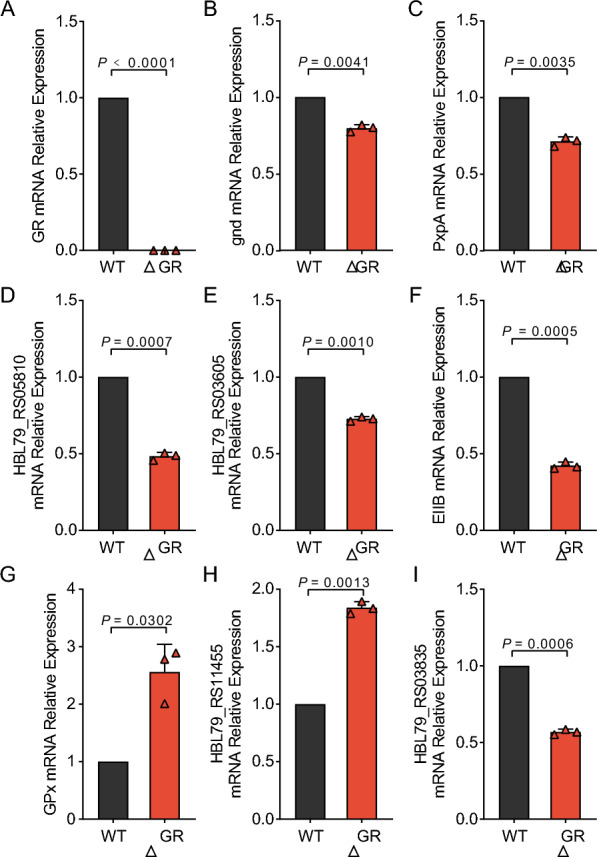
**Confirmation of oxidative stress-related differential gene expression changes in GR-knockout*Av. paragallinarum*by RT-qPCR. A** RT-qPCR was used to compare *GR* mRNA expression levels in wild-type (WT) and GR-knockout (ΔGR) strains (*P* < 0.0001). **B**–**I** RT-qPCR analysis of several metabolic genes including *gnd* (*P* < 0.0001) (**B**), *PxpA* (*P* = 0.0035) (**C**), *HBL79_RS05810* (*P* = 0.0005) (**D**), *HBL79_RS03605* (*P* = 0.0041) (**E**), *EIIB* (*P* = 0.0010) (**F**), GPx (*P* = 0.0007) (**G**), HBL79_RS11455 (*P* = 0.0302) (**H**) and HBL79_RS03835 (*P* = 0.0013) (**I**).

We next performed RT-qPCR to confirm that GR knockout led to transcriptomic alterations in *Av. paragallinarum*, particularly within the oxidative stress pathways. RT-qPCR analysis revealed that key metabolic genes, including *gnd*, *PxpA*, *HBL79_RS05810*, *HBL79_RS03605*, and *EIIB* were significantly downregulated, consistent with our transcriptomic results (Figures 4A–F). Notably, a significant decrease in the expression of *gnd*, which is pivotal for NADPH production and thus essential for maintaining cellular redox homeostasis and GSH levels, was observed in the GR mutant compared to the WT strain (Figure 4A), suggesting that GR knockout leads to important alterations in metabolic processes, particularly carboxyltransferase activity and carbohydrate metabolism, consistent with our earlier observations.

Conversely, genes implicated in the oxidative stress response and DNA modifications, such as those encoding *GPx* activity and *HBL79_RS11455*, a Class I SAM-dependent DNA methyltransferase, were upregulated, suggesting increased defense against oxidative damage and potential alterations in DNA methylation patterns that may facilitate the bacterium’s adaptation to increased oxidative stress conditions (Figures 4G and H). In addition, decreased expression of genes related to DNA replication and repair, notably *HBL79_RS03605* and *HBL79_RS03835*, was observed, indicating that the efficiency of DNA replication and repair mechanisms was reduced (Figure 4I). These findings are consistent with the observed decrease in growth rate and biosynthetic capacity in GR mutant strains and may reflect a broader reduction in biosynthetic pathways and cellular regulatory functions. Validation of these transcriptional shifts by RT-qPCR indicates that GR knockout leads to the strategic reconfiguration of metabolic and regulatory pathways in the bacterium. The downregulated genes suggest a reduction in biosynthetic activities and energy metabolism, while the upregulated genes indicate an adaptive response to oxidative stress. These findings not only corroborate our transcriptomic data but also further our understanding of the strategies employed by *Av. paragallinarum* for survival and genomic integrity under oxidative stress conditions.

### Energy metabolism shifts in GR-knockout *Av. paragallinarum*: an LC–MS/MS study

**Figure 5:**
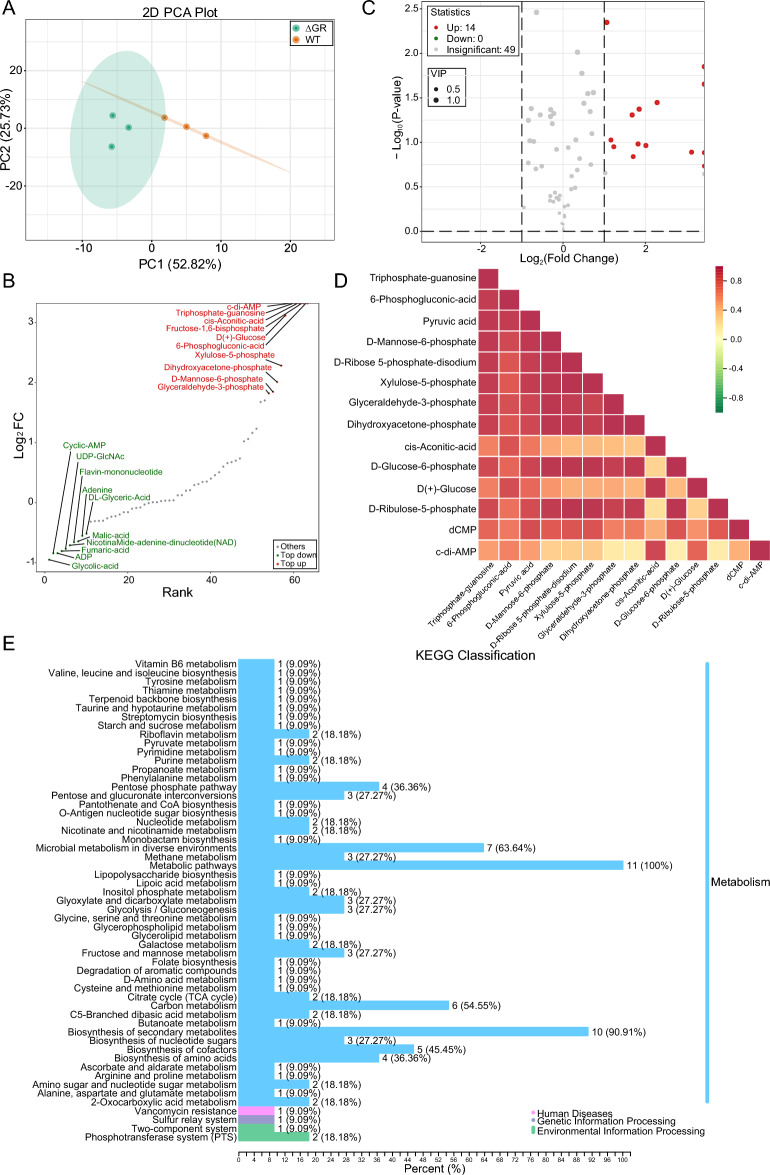
**LC–MS/MS metabolomics analysis unveils alterations in metabolic functions and pathways in *Av. paragallinarum*associated with GR knockout. A** PCA was used to compare the metabolic profiles of wild-type (WT) and GR knockout (ΔGR) strains of *Av. paragallinarum*. **B** Annotation of top differentially regulated metabolites. **C** Variable importance in projection (VIP) and fold change (FC) analysis of differentially regulated metabolites between WT and ΔGR strains. **D** Correlation heatmap displaying the coordinated metabolic response to GR knockout. **E** KEGG enrichment analysis of differentially regulated metabolites between WT and ΔGR strains of *Av. paragallinarum*.

Next, we examined the impact of alterations in energy metabolism using LC–MS/MS for an untargeted energy metabolomics analysis, which allowed us to capture a comprehensive snapshot of the organism’s metabolic state under modified redox conditions. Quality control measures confirmed the reliability of the metabolic data (Additional file 6). Metabolic profiling, enhanced by PCA and orthogonal partial least squares discriminant analysis (OPLS-DA), demonstrated a clear metabolomic divergence between the WT and GR mutant strains (Figure 5A), indicating the significant impact of GR knockout on the metabolic framework. Subsequent OPLS-DA identified 63 metabolites with significant differences between the two strains. Cluster analysis, visualized through a heatmap, and annotation of the top differentially regulated metabolites (Figure 5B) provided insights into the metabolic shifts. Specifically, a stringent selection based on variable importance in projection (VIP), fold change (FC), and statistical significance unveiled 14 significantly upregulated metabolites (Figure 5C), with patterns further explored in heatmaps and chord diagrams. These metabolites spanned across five key classes: Carbohydrate Metabolomics, Nucleotide and Its Metabolites, Organic Acids and Derivatives, Phosphate Sugars, and Phosphoric Acids, suggesting a metabolic realignment in response to the redox disequilibrium induced by the absence of GR. GR knockout seemed to affect these pathways particularly, hinting at a metabolic recalibration in response to the redox imbalance. The correlation heatmap and network of differential metabolites (Figure 5D) elucidated positive inter-metabolite correlations, indicating a coordinated metabolic adjustment to the GR knockout. KEGG classification and enrichment analysis (Figure 5E) revealed that the metabolic reconfigurations predominantly affected Metabolic Pathways, Biosynthesis of Secondary Metabolites, and Microbial Metabolism in Diverse Environments, highlighting the extensive influence of GR on the bacterium’s metabolic processes. Further details and supplementary analyses can be found in Additional file 7.

In summary, our comprehensive energy metabolomics study highlights the indispensable role of GR in modulating the metabolic functions of *Av. paragallinarum*. The uniform upregulation of important metabolites underscores a potential compensatory adaptation by the bacterium to maintain viability under the challenges of altered metabolic and oxidative states. These findings not only enrich our genetic understanding but also broaden our comprehension of the metabolic and physiological adaptations of *Av. paragallinarum* in facing internal redox perturbations.

### Profiling of redox metabolites in GR-knockout *Av. Paragallinarum*

**Figure 6:**
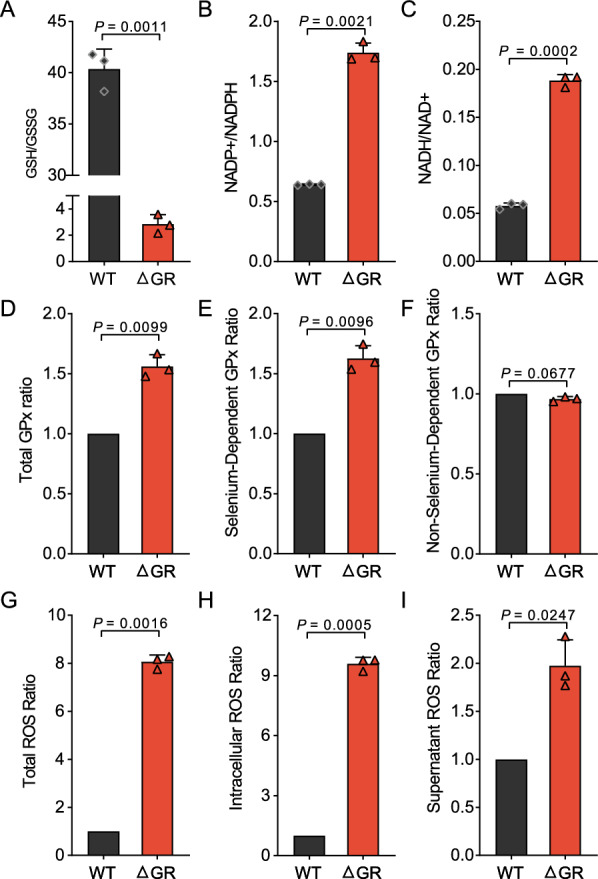
**Analysis of oxidative stress metabolites in*Av. paragallinarum*following GR knockout. A** GSH/GSSG ratio in wild-type (WT) and GR-deficient (ΔGR) strains of *Av. paragallinarum* (*P* = 0.0011). **B** NADP + /NADPH ratio in WT and ΔGR strains (*P* = 0.0021). **C.** NADH/NAD + ratio in WT and ΔGR strains (*P* = 0.0002). **D** Total GPx ratio in WT and ΔGR strains (*P* = 0.0099). **E** Selenium-dependent GPx ratio in WT and ΔGR strains (*P* = 0.0096). **F** Non-selenium-dependent GPx ratio in WT and ΔGR strains (*P* = 0.0677). **G** Total ROS ratio in WT and ΔGR strains (*P* = 0.0016). **H** Intracellular ROS ratio in WT and ΔGR strains (*P* = 0.0005). **I** Supernatant ROS ratio in WT and ΔGR strains (*P* = 0.0247).

Next, we examined redox metabolome alterations induced by GR knockout in *Av. paragallinarum* to further understand the role of these genetic modifications in mediating endogenous oxidative stress and redox balance, which are important aspects of bacterial physiology and stress management. We found a significant reduction in the GSH/GSSG ratio in the GR-deficient strain compared to the WT strain (*P* = 0.0011), indicating severe oxidative stress (Figure 6A). This was accompanied by an elevated NADP^+^/NADPH ratio (*P* = 0.0021) (Figure 6B), suggesting a decrease in the utilization of NADPH during the reduction of GSSG to GSH. Concurrently, a significant increase in NADH/NAD^+^ ratio (*P* = 0.0002) was observed (Figure 6C), suggesting that the observed metabolic disruptions were a result of oxidative stress, potentially as a compensatory mechanism to sustain energy production and redox homeostasis in the absence of GR. Intriguingly, a significant increase in total and selenium-dependent GPx ratios was observed (*P* = 0.0099 and *P* = 0.0096, respectively) (Figures 6D and E), indicating an adaptive upregulation to mitigate oxidative damage, while the non-selenium-dependent GPx ratio remained stable (Figure 6F). This stability may reflect a distinct regulatory scheme or a lesser role in countering the redox state alterations induced by GR knockout. Moreover, ROS levels, pivotal markers of oxidative stress, surged post-GR knockout, across total, intracellular, and supernatant ROS ratios (*P* = 0.0016, *P* = 0.0005, and *P* = 0.0247, respectively) (Figures 6G–I), with intracellular elevations being particularly significant. These increases underline the broad impact of GR deletion on the oxidative stress status in the bacterium, which primarily affect intrinsic oxidative stress mechanisms.

Together, our findings identify a significant redox imbalance triggered by GR knockout in *Av. paragallinarum*, reflected by shifts in key redox-sensitive metabolite levels and altered GPx activities and ROS levels. Our redox metabolitic profiling data not only validate our earlier genetic and metabolic insights but also enhance our comprehension of the metabolic and physiological adaptations to internal redox challenges in *Av. paragallinarum*, revealing the complex interplay of redox regulation in bacterial survival and the stress response.

## Discussion

GR serves as a cornerstone in cellular defense mechanisms against oxidative stress, safeguarding redox homeostasis essential for bacterial survival and function [26]. Although the pivotal roles of GR in higher organisms have been well-documented [27, 28], its specific contributions and underlying mechanisms within NAD-dependent bacteria such as *Av. paragallinarum* remain unclear. This study addressed this gap in the literature, detailing the regulatory effects of GR on internal oxidative stress within *Av. paragallinarum*. Using this bacterium as a model, we identified a role for GR in intricately managing the balance of GSH and GSSG, which is essential for neutralizing ROS and protecting against oxidative cellular damage. This equilibrium transcends biochemical necessity and is a crucial factor for the survival of the bacterium against environmental and host-induced stresses [29]. Previous studies demonstrating that GR knockout increases the sensitivity of *Acidithiobacillus caldus* to heavy metals further support our findings [30]. Our comprehensive analyses demonstrated that GR deficiency leads to a significant disruption in redox homeostasis, evidenced by altered GSH/GSSG and NADP + /NADPH ratios, underscoring the indispensable role of GR in maintaining the oxidative stress defense system. Furthermore, we constructed a comprehensive overview diagram that delineates the multifaceted pathways through which GR deficiency mediates the regulation of the bacterial internal oxidative stress network. This illustration captures the intricate web of interactions and adjustments the bacterium employs to navigate the challenges posed by oxidative stress in the absence of GR (Additional file 8). These findings not only extend our understanding of the function of GR in bacterial metabolic processes and virulence mechanisms but also highlight its significance in the broader context of microbial pathogenicity and stress resistance mechanisms.

The absence of GR in *Av. paragallinarum* not only disrupts oxidative stress management but also significantly impacts bacterial physiology and virulence. Through our in vivo and in vitro studies, we demonstrated that GR deficiency leads to a pronounced reduction in bacterial growth and biofilm formation, and a weakened interaction with host macrophages. These findings highlight the essential role of GR in mechanisms of bacterial pathogenicity. The reduction in biofilm formation, which is closely associated with the attenuation of bacterial virulence and diminished resistance to host defenses [31, 32], suggests that GR plays a important determinant beyond the oxidative stress response, influencing the ability of the bacterium to establish and maintain infections. Furthermore, the interaction between *Av. paragallinarum* and host macrophages plays a crucial role in determining bacterial virulence. The weakened interaction observed in GR-deficient strains indicates a reduced capacity to evade or resist host immune responses, which is important for establishing and maintaining infections. This aligns with previous studies showing that effective macrophage interaction is essential for the virulence of various bacterial pathogens. The diminished ability to adhere to and invade macrophages likely contributes to the overall attenuation of virulence observed in the GR knockout strains, highlighting the multifaceted role of GR in bacterial pathogenicity [33, 34]. Interestingly, the reduction in virulence among GR-knockout *Av. paragallinarum* strains reveals an important insight: the ability of the bacterium to cause disease depends on managing oxidative stress. Recently, Zhang et al. reported that disrupting GPx heightened both oxidative stress resilience and virulence in *Listeria monocytogenes* [35]. Similarly, sRNA was shown to modulate the oxidative stress response and virulence in *Xanthomonas oryzae pv. Oryzicola* [36], suggesting a profound connection between oxidative stress management and pathogenicity. Together, these studies suggest that the ability of bacterium to thrive in the hostile environment of the host is significantly compromised in the absence of GR, emphasizing the role of GR in facilitating a successful infection. In light of these findings, it is evident that the role of GR in the pathogenicity of *Av. paragallinarum* is profound, affecting not only its survival under oxidative stress but also its ability to colonize and cause disease in the host. Additionally, we acknowledge that GR is not the only enzyme involved in the oxidative stress response in *Av. paragallinarum*. The genome of *Av. paragallinarum* also contains other oxidative stress-related genes, such as catalase (*katA*), superoxide dismutase (*sodA*), and peroxidase (*prx*), which may complement the role of GR [37–39]. These enzymes likely contribute to the overall oxidative stress defense system, providing redundancy and robustness to the bacterium’s ability to manage oxidative stress. Further investigation into these genes will help to build a more comprehensive understanding of the oxidative stress response in *Av. paragallinarum* and how GR interacts with other components of this defense system.

The dual function of GR in modulating both oxidative stress defense and pathogenic traits suggests that GR could potentially serve as a novel antimicrobial target. The dependency of *Av. paragallinarum* on GR for oxidative stress mitigation and virulence maintenance not only emphasizes its vital role in bacterial survival but also suggests that impairing GR function could significantly reduce pathogenicity. We believe that this vulnerability opens innovative avenues in bacterial infection control and propose that inhibiting GR activity or expression may weaken bacterial oxidative defenses, thereby reducing their virulence. Specifically targeting bacterial redox processes through inhibition of GR capitalizes on a unique aspect of bacterial defense. Inhibition of GR disrupts the GSH/GSSG balance, leading to the accumulation of ROS and subsequent oxidative damage beyond the repair capability of the bacterium. The application of GR inhibitors, already reported to have positive outcomes for tumor, cancer, and parasite control [40–42], shows promise for a broad range of bacteria due to their universal reliance on GR for managing oxidative stress. Developing GR inhibitors as antimicrobial agents could enhance current antibiotic strategies, potentially circumventing existing resistance issues. Focusing on a fundamental process like redox balance, GR inhibitors may offer a distinct mode of action compared to traditional antibiotics, thereby reducing the risk of cross-resistance. However, advancing GR as an antimicrobial target necessitates addressing potential challenges, such as ensuring the specificity of bacterial GR inhibitors to avoid affecting host cells. Future efforts should therefore concentrate on identifying potent, selective GR inhibitors, elucidating their mechanisms, and affirming their effectiveness and safety in preclinical studies. Ultimately, targeting GR paves the way for innovative antimicrobial strategies, exploiting its pivotal role in bacterial oxidative stress management and connection to virulence to introduce a novel class of antimicrobial agents. This approach not only expands our antimicrobial toolkit but also underscores the importance of redox biology in understanding and targeting bacterial pathogenicity.

## Supplementary Information

**Additional file 1. Strains used in this study**.

**Additional file 2. Vectors used in this study**.

**Additional file 3. Primers used in this study**. Note: The blue nucleotides represent the restriction enzyme sites (Eco RI, BamHI), the red nucleotides indicate the USS sequence, and the orange segment denotes the KanR overlap region.

**Additional file 4. Quality control analysis of transcriptomic sequencing data for *Av. paragallinarum***. A Distribution of sequencing error rates for WT and GR-knockout (ΔGR) strains of *Av. paragallinarum*. The x-axis represents the position of bases in reads, and the y-axis indicates the sequencing error rate, with a division between Read 1 and Read 2 marked by a dashed vertical line. B Base content distribution across sequencing reads. The x-axis denotes base position in reads, while the y-axis displays the percentage of each base type, with Read 1 and Read 2 delineated by a dashed vertical line. C Saturation curves illustrating the proportion of genes with quantification errors within 15% relative to the proportion of data extracted. Each curve color denotes a different FPKM quantification level. D Correlation matrix and scatter plots assessing RNA-Seq reproducibility between samples. Matrix cells and corresponding scatter plot points represent squared correlation coefficients, reflecting the robustness of transcriptomic relationships across samples.

**Additional file 5. Transcriptomic characteristics and functional annotation before and after GR knockout in *Av. paragallinarum***. A Identification of 211 DEGs including123 upregulated and 88 downregulated genes. B GO enrichment analysis of the DEGs shown as a circos plot. C KEGG pathway enrichment analysis of the DEGs shown as a circus plot. D, E Detailed GO enrichment analysis of the upregulated DEGs (D) and downregulated DEGs (E).

**Additional file 6. LC–MS/MS quality control and principal component analysis of energy metabolomics in *Av. paragallinarum***. A Total Ion Current (TIC) Overlapping Profiles. Metabolite detection exhibits high overlapping of total ion flow curves, indicating consistent retention times and peak intensities, demonstrating signal stability in mass spectrometry across different testing times for the same sample. The high instrument stability is crucial for the repeatability and reliability of the data. B QC Sample Correlation Analysis. Diagonal squares represent QC sample names; the lower left scatter plots against the diagonal show correlations with metabolite levels on the axes, with each point representing a metabolite; the upper right squares contain corresponding QC sample correlation coefficients. C CV Distribution Across Sample Groups. The x-axis represents CV values, and the y-axis indicates the proportion of substances with less than the corresponding CV value relative to the total number of substances. Different colors represent different sample groups, with QC denoting quality control samples. Vertical reference lines correspond to CV values of 0.2 and 0.3, and the horizontal reference line represents 80% of the total number of substances. D, E PCA Score Plots for All Group Samples. All PCA ellipse (D) and all no-QC PCA 3D (E). PC1, PC2, and PC3 represent the first, second, and third principal components, respectively, with percentages indicating the variance explained by each component. Each point represents a sample, with the same color denoting samples from the same group; Group represents the grouping. F Overall Sample PC1 Control Chart. The chart’s x-axis is the sample detection order, and the y-axis reflects the PC1 value, with yellow and red lines defining ranges for positive and negative two and three standard deviations, respectively. Green points represent QC samples, and black points denote experimental samples.

**Additional file 7. LC–MS/MS metabolomics analysis unveils alterations in metabolic functions and pathways in *Av. paragallinarum* associated with GR knockout**. A OPLS-DA plot showing significant metabolic differences between WT and ΔGR strains. B Heatmap showing the cluster analysis of metabolite levels in WT and ΔGR strains. C Heatmap analysis of the 14 identified upregulated metabolites. D Chord diagram showing the metabolic pathways and the interconnectivity of the significantly altered metabolites in the ΔGR strain. E Network of differential metabolites displaying the coordinated metabolic response to GR knockout. F KEGG classification analysis of differentially regulated metabolites between WT and ΔGR strains of *Av. paragallinarum*.

**Additional file 8: Overview of metabolic pathway alterations and ribosomal protein expression changes in *Av. paragallinarum* following GR knockout**. The diagram depicts a comprehensive map of metabolic processes with enzymes coded by differentially expressed genes (DEGs) following the GR gene disruption. Blue boxes represent enzymes associated with metabolic pathways that were not detected; green boxes indicate enzymes that were detected but did not show significant expression changes; red boxes signify downregulated enzymes, and purple boxes signify upregulated enzymes. In addition, upregulation in ribosomal proteins is denoted by pink shading in the ribosomal structure to the right. The metabolic pathways and corresponding enzymes are organized around the TCA cycle at the center, branching out to include amino acid metabolism, purine metabolism, and the glutathione cycle, highlighting the interconnectivity and complex response to the oxidative stress induced by the absence of GR.

## Data Availability

The datasets used and/or analysed during the current study are available from the corresponding author upon reasonable request.

## Abbreviations

CFU: colony-forming unit
DEG: differentially expressed gene
GPx: glutathione peroxidase
GR: glutathione reductase
GSH: reduced glutathione
GSSG: oxidized glutathione
LC–MS/MS: liquid chromatography-mass spectrometry/mass spectrometry
NAD^+^: nicotinamide adenine dinucleotide (oxidized form)
NADH: nicotinamide adenine dinucleotide (reduced form)
NADP^+^: nicotinamide adenine dinucleotide phosphate (oxidized form)
NADPH: nicotinamide adenine dinucleotide phosphate (reduced form)
PCR: polymerase chain reaction
ROS: reactive oxygen species
RT-qPCR: quantitative reverse transcription polymerase chain reaction
WT: wild-type
