# Correction: Glutathione reductase modulates endogenous oxidative stress and affects growth and virulence in *Avibacterium paragallinarum*

**DOI:** 10.1186/s13567-025-01499-8

**Published:** 2025-03-21

**Authors:** Yan Zhi, Chen Mei, Zhenyi Liu, Ying Liu, Hongjun Wang

**Affiliations:** Institute of Animal Husbandry and Veterinary Medicine, Beijing Academy of Agriculture and Forestry Sciences, Beijing, China

**Correction: Veterinary Research (2025) 56:1** 10.1186/s13567-024-01388-6

Following publication of the original article [1], the authors identified an error in the Funding section.

The incorrect Funding is:

This research was supported by the Beijing Natural Science Foundation (6212009), and the Reform and Development Project of Beijing Academy of Agricultural and Forestry Sciences (XMS202411).

The correct Funding is:

This work was supported by the Special Science and Technology Research Project for Innovation Capacity Building of Beijing Academy of Agriculture and Forestry Sciences (Research and Development of New Inputs and Innovative Technologies for Integrated Prevention and Control of Major Animal Diseases), and the Reform and Development Project of Beijing Academy of Agricultural and Forestry Sciences (XMS202411, XMS202510).
