# Supplementary material for: Glutathione reductase modulates endogenous oxidative stress and affects growth and virulence in Avibacterium paragallinarum
Source: Vet Res. 2025 Jan 2;56:1. doi: 10.1186/s13567-024-01388-6 (PMC11697956; doi:10.1186/s13567-024-01388-6)
Supplement: Supplementary file 1 — Additional file 1. Strains used in this study. [file 13567_2024_1388_MOESM1_ESM.docx]

**Additional file 1 Strains used in this study**

|  | |  | |  |
| --- | --- | --- | --- | --- |
| **Strains** | **Description** | | **Source** | |
| *E. coli DH5α* | General cloning and plasmid maintenance strain | | Purchased from TransGen Biotech | |
| *Av. paragallinarum Modesto* | Protein expression strain | | Lab collection | |
