# Supplementary material for: Glutathione reductase modulates endogenous oxidative stress and affects growth and virulence in Avibacterium paragallinarum
Source: Vet Res. 2025 Jan 2;56:1. doi: 10.1186/s13567-024-01388-6 (PMC11697956; doi:10.1186/s13567-024-01388-6)
Supplement: Supplementary file 2 — Additional file 2. Vectors used in this study. [file 13567_2024_1388_MOESM2_ESM.docx]

**Additional file 2 Vectors used in this study**

|  | | |
| --- | --- | --- |
| **Plasmids** | **Description** | **Source** |
| pK18mobsacB | Suicide vector used for natural transformation | This study |
| pET28a | Vector used for cloning the kanamycin resistance cassette gene | This study |
