# Supplementary material for: Glutathione reductase modulates endogenous oxidative stress and affects growth and virulence in Avibacterium paragallinarum
Source: Vet Res. 2025 Jan 2;56:1. doi: 10.1186/s13567-024-01388-6 (PMC11697956; doi:10.1186/s13567-024-01388-6)
Supplement: Supplementary file 3 — Additional file 3. Primers used in this study. Note: The blue nucleotides represent the restriction enzyme sites (Eco RI, BamHI), the red nucleotides indicate the USS sequence, and the orange segment denotes the KanR overlap region. [file 13567_2024_1388_MOESM3_ESM.docx]

**Additional file 3 primers used in this study**

| **Primer name** | **Primer sequence (5' - 3')** | **length** |
| --- | --- | --- |
| kan-F | ATGAGCCATATTCAACGGGAAACG | 816bp |
| kan-R | TTAGAAAAACTCATCGAGCATCAAAT |  |
| gr-up-EcoRI-F | CCGGAATTCAAGTGCGGTACGGTTTTCAGCAAATTAAAGC | 534bp |
| gr-up-kan-R | TCCCGTTGAATATGGCTCATTTTTAACTCCCTAAATTGGTGAATTGG |  |
| gr-kan-down-F | TGCTCGATGAGTTTTTCTAATATTAAGTGCGGTGTAAATTTTCAT | 513bp |
| gr-down-BamHI-R | CGCGGATCCACCGCACTTTAATATTCGCCACCGTGCC |  |
| GR-F | ATGACAAAACATTATGATTAC | 1356bp |
| GR-R | TTAACGCATTGTTACAAATTC |  |
| gr-F | ATCGCAACAGGGGCTTATCC | 173bp |
| gr-R | CAACCGCAACGGATTTTGGT |  |
| gnd-F | GCAGGACAAAAAGGCACAGG | 145bp |
| gnd-R | GCAACGGGCAAACACAGATT |  |
| pxpA-F | GCTTGTGGTTTACACGCAGG | 119bp |
| pxpA-R | AGCCCCAACTCGCACATTAT |  |
| RS05810-F | CGCTTGTGTTATTGAGGCGG | 183bp |
| RS05810-R | GATAACGTTCGCGTGCTTCC |  |
| RS03605-F | ACATCAGCTCCCTCCAAGAGA | 157bp |
| RS03605-R | CCAATGTAGCAATCGCCTCC |  |
| EIIB-F | ATCGGCGTTTTAGTTGGGGT | 151bp |
| EIIB-R | GCCAACGGGGATAAAATCGC |  |
| GPx-F | GCGAAAGCGAAAGGGTTGTT | 114bp |
| GPx-R | CGCTACCACCGATGTACTCC |  |
| recF-F | ATGGGCGAATCCAAGAGCAA | 181bp |
| recF-R | GGGTGATGATTTGCATCGGC |  |
| RS11455-F | TCCCAACGGGATTGTTCTGG | 190bp |
| RS11455-R | TACCAAGCCTTGCTCCACAG |  |
| RS03835-F | GGTTCAAAGCAAATGGCGGT | 144bp |
| RS03835-R | TGAATGAAGACCACCCAGCC |  |
| AgaS-F | GGGGCATTCCTTACAGCCTT | 103bp |
| AgaS-R | AGGGCTGTTACCTGAACGTG |  |

Note: The blue nucleotides represent the restriction enzyme sites (Eco RI, BamHI), the red nucleotides indicate the USS sequence, and the orange segment denotes the KanR overlap region.
