# Supplementary figures and images for: Glutathione reductase modulates endogenous oxidative stress and affects growth and virulence in Avibacterium paragallinarum
Source: Vet Res. 2025 Jan 2;56:1. doi: 10.1186/s13567-024-01388-6 (PMC11697956; doi:10.1186/s13567-024-01388-6)

A

WT vs ΔGR

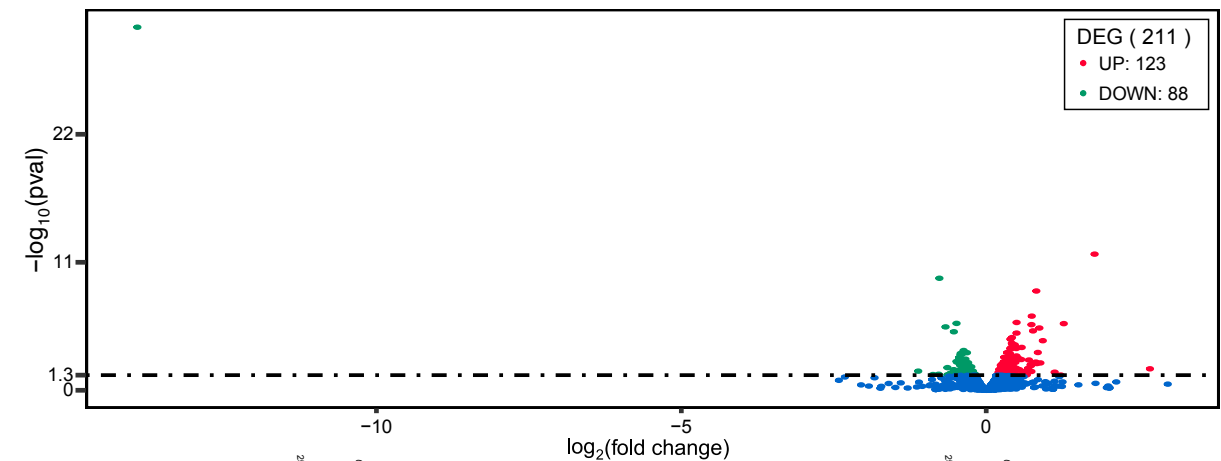

B

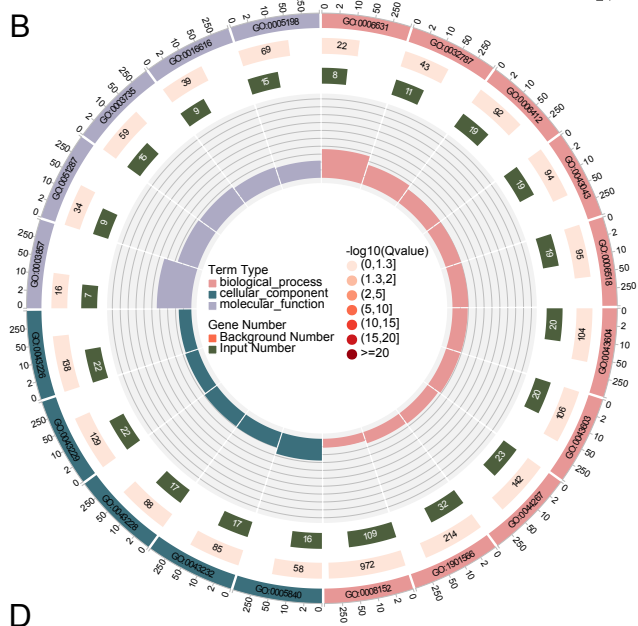

C

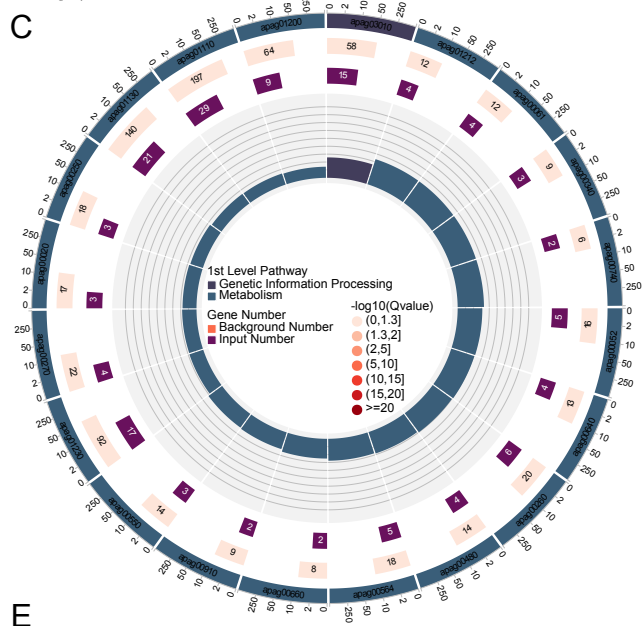

D

The Most Enriched GO Terms (WT vs ΔGR\_UP)

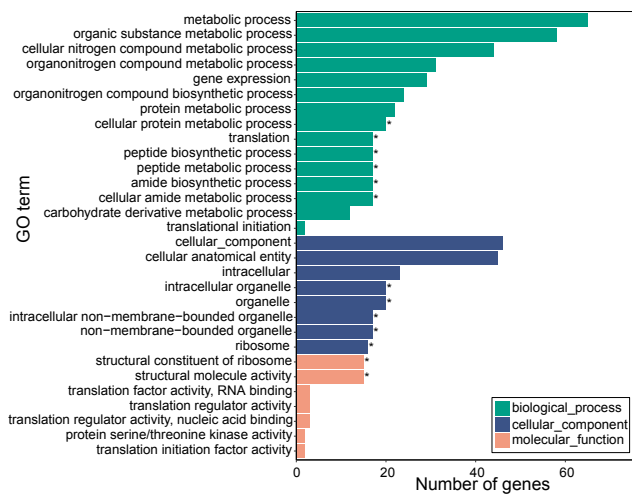

E

The Most Enriched GO Terms (WT vs ΔGR\_DOWN)

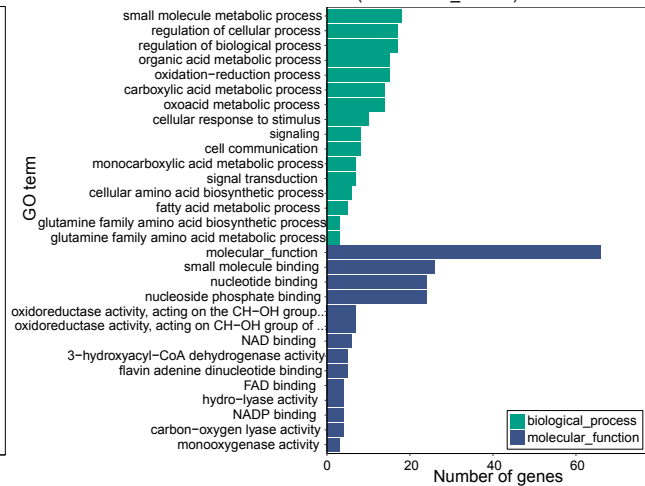

Supplement: Supplementary file 4 — Additional file 4. Quality control analysis of transcriptomic sequencing data for Av. paragallinarum. A Distribution of sequencing error rates for WT and GR-knockout (ΔGR) strains of Av. paragallinarum. The x-axis represents the position of bases in reads, and the y-axis indicates the sequencing error rate, with a division between Read 1 and Read 2 marked by a dashed vertical line. B Base content distribution across sequencing reads. The x-axis denotes base position in reads, while the y-axis displays the percentage of each base type, with Read 1 and Read 2 delineated by a dashed vertical line. C Saturation curves illustrating the proportion of genes with quantification errors within 15% relative to the proportion of data extracted. Each curve color denotes a different FPKM quantification level. D Correlation matrix and scatter plots assessing RNA-Seq reproducibility between samples. Matrix cells and corresponding scatter plot points represent squared correlation coefficients, reflecting the robustness of transcriptomic relationships across samples. [file 13567_2024_1388_MOESM4_ESM.pdf]

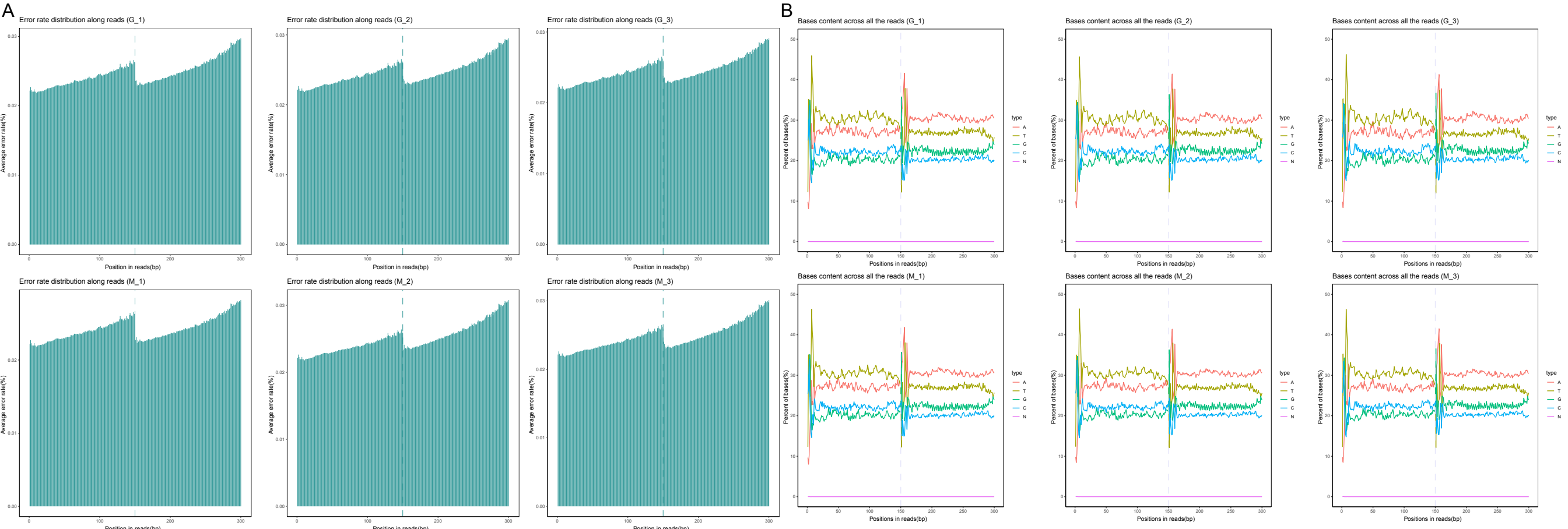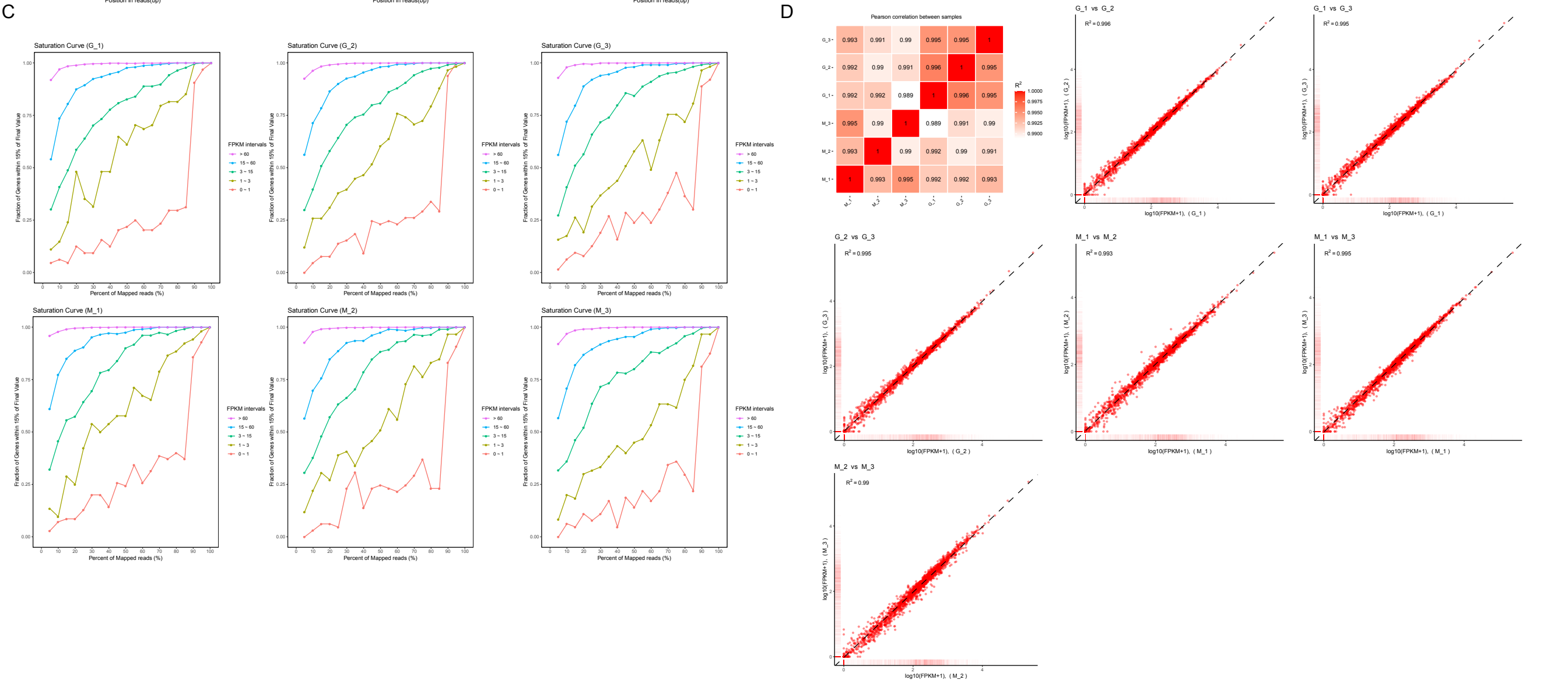

Supplement: Supplementary file 5 — Additional file 5. Transcriptomic characteristics and functional annotation before and after GR knockout in Av. paragallinarum. A Identification of 211 DEGs including123 upregulated and 88 downregulated genes. B GO enrichment analysis of the DEGs shown as a circos plot. C KEGG pathway enrichment analysis of the DEGs shown as a circus plot. D, E Detailed GO enrichment analysis of the upregulated DEGs (D) and downregulated DEGs (E). [file 13567_2024_1388_MOESM5_ESM.pdf]

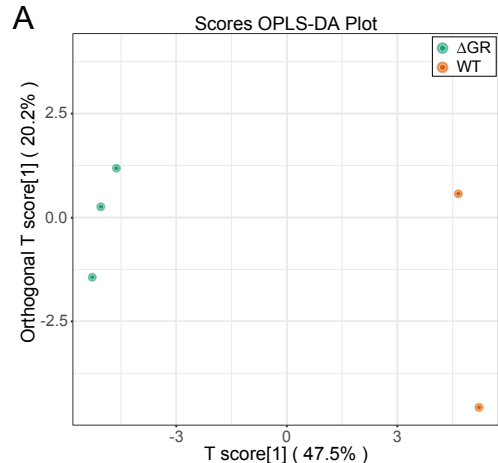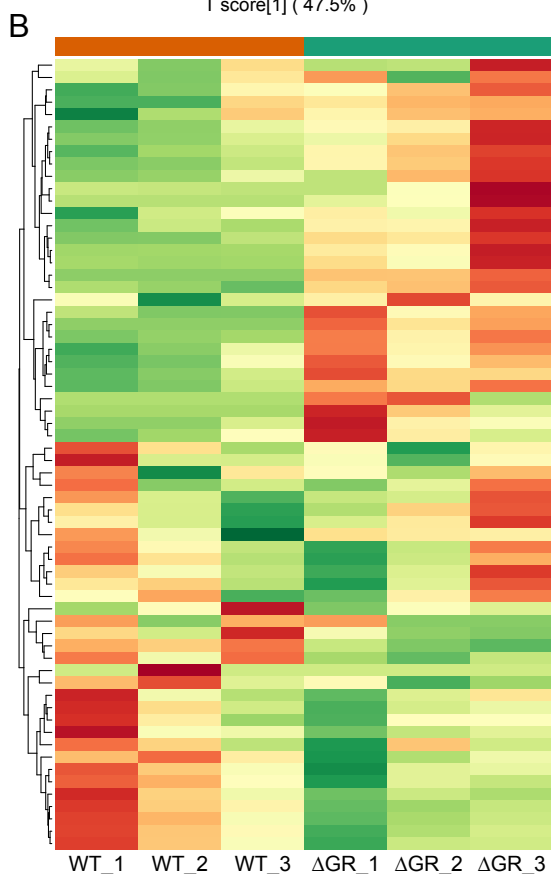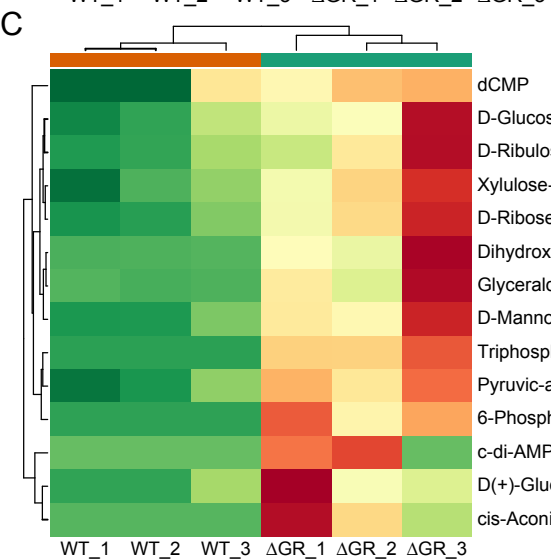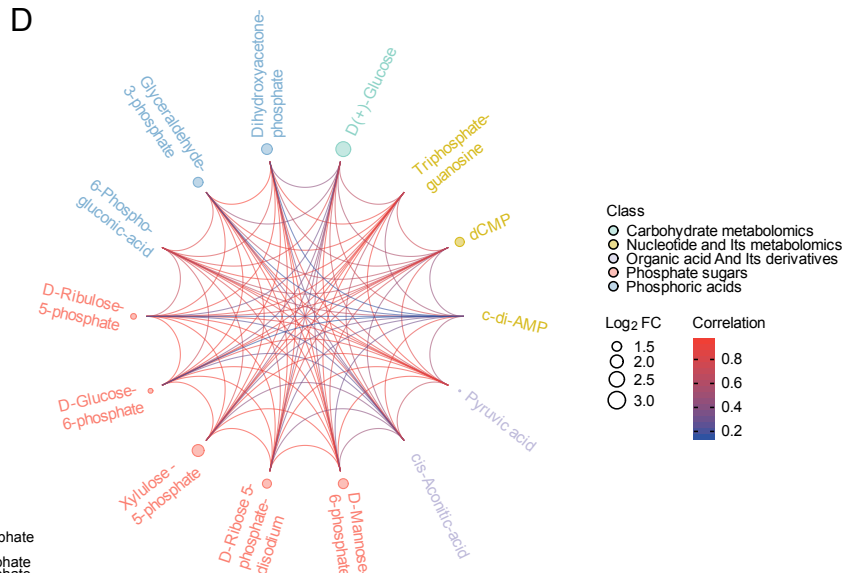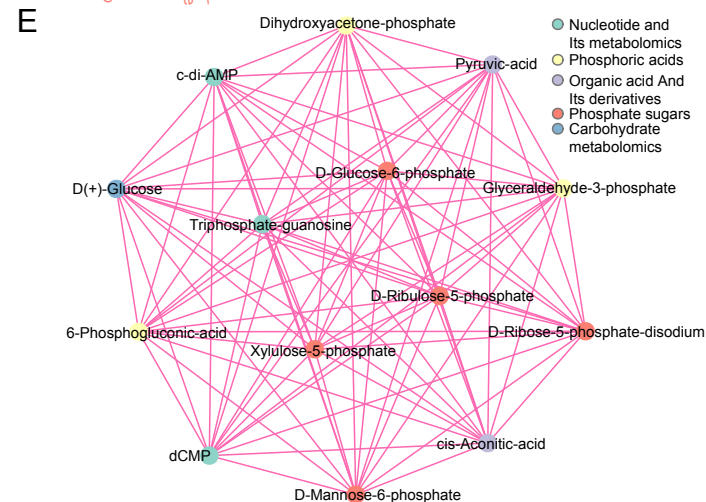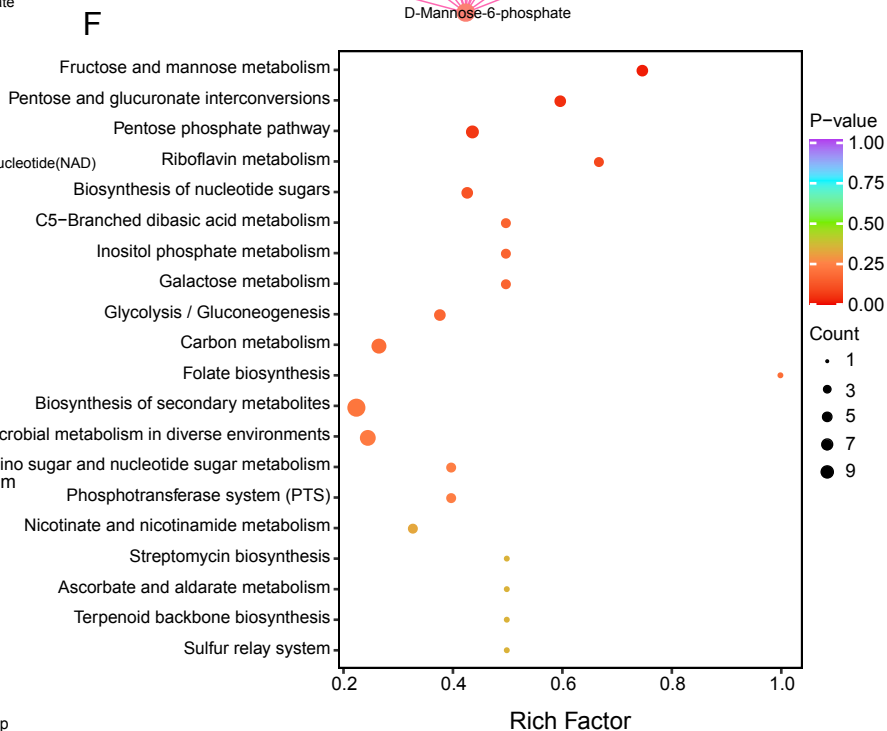

Supplement: Supplementary file 7 — Additional file 7. LC–MS/MS metabolomics analysis unveils alterations in metabolic functions and pathways in Av. paragallinarum associated with GR knockout. A OPLS-DA plot showing significant metabolic differences between WT and ΔGR strains. B Heatmap showing the cluster analysis of metabolite levels in WT and ΔGR strains. C Heatmap analysis of the 14 identified upregulated metabolites. D Chord diagram showing the metabolic pathways and the interconnectivity of the significantly altered metabolites in the ΔGR strain. E Network of differential metabolites displaying the coordinated metabolic response to GR knockout. F KEGG classification analysis of differentially regulated metabolites between WT and ΔGR strains of Av. paragallinarum. [file 13567_2024_1388_MOESM7_ESM.pdf]
