# Supplementary material for: Glutathione reductase modulates endogenous oxidative stress and affects growth and virulence in Avibacterium paragallinarum
Source: Vet Res. 2025 Jan 2;56:1. doi: 10.1186/s13567-024-01388-6 (PMC11697956; doi:10.1186/s13567-024-01388-6)
Supplement: Supplementary file 6 — Additional file 6. LC–MS/MS quality control and principal component analysis of energy metabolomics in Av. paragallinarum. A Total Ion Current (TIC) Overlapping Profiles. Metabolite detection exhibits high overlapping of total ion flow curves, indicating consistent retention times and peak intensities, demonstrating signal stability in mass spectrometry across different testing times for the same sample. The high instrument stability is crucial for the repeatability and reliability of the data. B QC Sample Correlation Analysis. Diagonal squares represent QC sample names; the lower left scatter plots against the diagonal show correlations with metabolite levels on the axes, with each point representing a metabolite; the upper right squares contain corresponding QC sample correlation coefficients. C CV Distribution Across Sample Groups. The x-axis represents CV values, and the y-axis indicates the proportion of substances with less than the corresponding CV value relative to the total number of substances. Different colors represent different sample groups, with QC denoting quality control samples. Vertical reference lines correspond to CV values of 0.2 and 0.3, and the horizontal reference line represents 80% of the total number of substances. D, E PCA Score Plots for All Group Samples. All PCA ellipse (D) and all no-QC PCA 3D (E). PC1, PC2, and PC3 represent the first, second, and third principal components, respectively, with percentages indicating the variance explained by each component. Each point represents a sample, with the same color denoting samples from the same group; Group represents the grouping. F Overall Sample PC1 Control Chart. The chart’s x-axis is the sample detection order, and the y-axis reflects the PC1 value, with yellow and red lines defining ranges for positive and negative two and three standard deviations, respectively. Green points represent QC samples, and black points denote experimental samples. [file 13567_2024_1388_MOESM6_ESM.pdf]

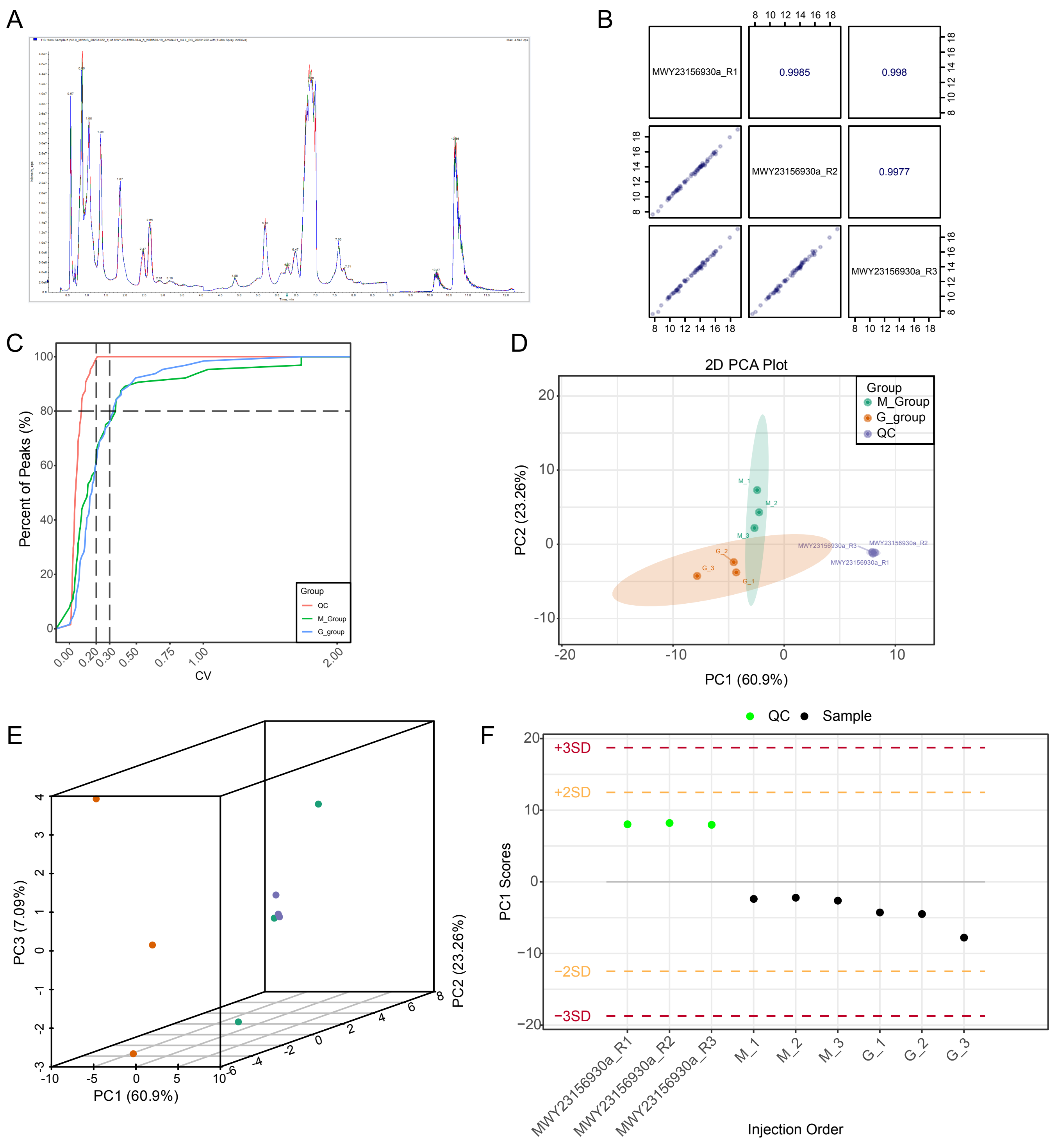
A
B
MWY23156930a_R1
0.9985
0.998
MWY23156930a_R2
0.9977
MWY23156930a_R3
C
Percent of Peaks (%)
CV
Group
QC
M_Group
G_group
D
2D PCA Plot
PC1 (60.9%)
PC2 (23.26%)
Group
M_Group
G_group
QC
E
PC1 (60.9%)
PC2 (23.26%)
PC3 (7.09%)
F
QC
Sample
+3SD
+2SD
−2SD
−3SD
PC1 Scores
Injection Order
MWY23156930a_R1
MWY23156930a_R2
MWY23156930a_R3
M_1
M_2
M_3
G_1
G_2
G_3
