# Supplementary material for: Glutathione reductase modulates endogenous oxidative stress and affects growth and virulence in Avibacterium paragallinarum
Source: Vet Res. 2025 Jan 2;56:1. doi: 10.1186/s13567-024-01388-6 (PMC11697956; doi:10.1186/s13567-024-01388-6)
Supplement: Supplementary file 8 — Additional file 8: Overview of metabolic pathway alterations and ribosomal protein expression changes in Av. paragallinarum following GR knockout. The diagram depicts a comprehensive map of metabolic processes with enzymes coded by differentially expressed genes (DEGs) following the GR gene disruption. Blue boxes represent enzymes associated with metabolic pathways that were not detected; green boxes indicate enzymes that were detected but did not show significant expression changes; red boxes signify downregulated enzymes, and purple boxes signify upregulated enzymes. In addition, upregulation in ribosomal proteins is denoted by pink shading in the ribosomal structure to the right. The metabolic pathways and corresponding enzymes are organized around the TCA cycle at the center, branching out to include amino acid metabolism, purine metabolism, and the glutathione cycle, highlighting the interconnectivity and complex response to the oxidative stress induced by the absence of GR. [file 13567_2024_1388_MOESM8_ESM.pdf]

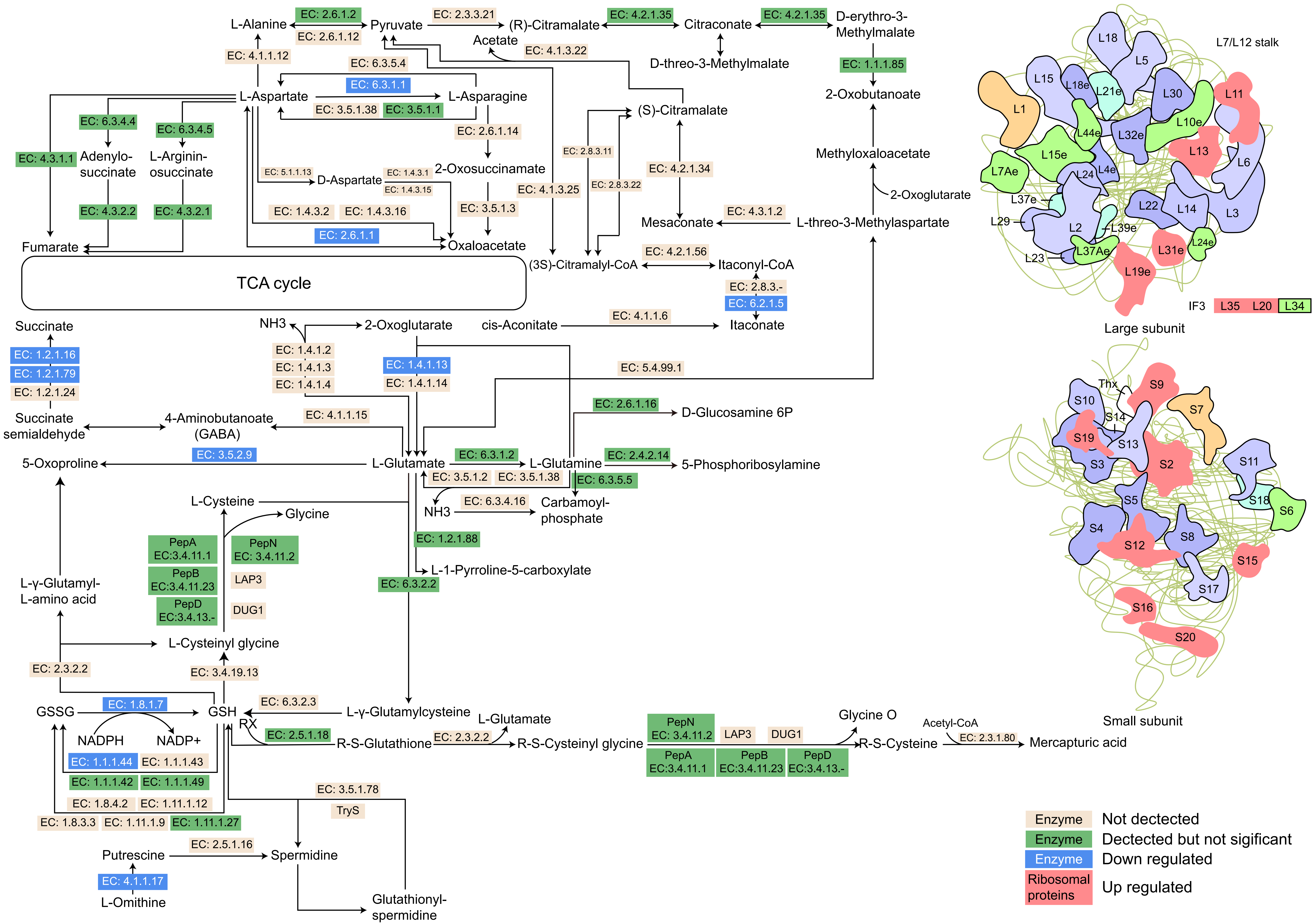

L-Alanine
EC: 2.6.1.2
EC: 2.6.1.12
Pyruvate
EC: 2.3.3.21
(R)-Citramalate
EC: 4.2.1.35
Citraconate
EC: 4.2.1.35
D-erythro-3-Methylmalate
Acetate
EC: 4.1.3.22
D-threo-3-Methylmalate
EC: 1.1.1.85
2-Oxobutanoate
EC: 4.1.1.12
EC: 6.3.5.4
EC: 6.3.1.1
L-Aspartate
L-Asparagine
EC: 3.5.1.38
EC: 3.5.1.1
(S)-Citramalate
EC: 6.3.4.4
EC: 6.3.4.5
EC: 2.6.1.14
EC: 4.3.1.1
Adenylo-succinate
L-Arginin-osuccinate
EC: 2.8.3.11
EC: 4.2.1.34
Methyloxaloacetate
EC: 5.1.1.13
D-Aspartate
EC: 1.4.3.1
EC: 1.4.3.15
2-Oxosuccinamate
EC: 4.1.3.25
EC: 2.8.3.22
2-Oxoglutarate
EC: 4.3.2.2
EC: 4.3.2.1
EC: 1.4.3.2
EC: 1.4.3.16
EC: 3.5.1.3
Mesaconate
EC: 4.3.1.2
L-threo-3-Methylaspartate
EC: 2.6.1.1
Fumarate
Oxaloacetate
EC: 4.2.1.56
TCA cycle
(3S)-Citramalyl-CoA
Itaconyl-CoA
EC: 2.8.3.-
EC: 6.2.1.5
Succinate
NH3
2-Oxoglutarate
cis-Aconitate
EC: 4.1.1.6
Itaconate
EC: 1.2.1.16
EC: 1.2.1.79
EC: 1.2.1.24
EC: 1.4.1.2
EC: 1.4.1.3
EC: 1.4.1.4
EC: 1.4.1.13
EC: 1.4.1.14
EC: 5.4.99.1
Succinate semialdehyde
4-Aminobutanoate (GABA)
EC: 4.1.1.15
EC: 2.6.1.16
D-Glucosamine 6P
EC: 3.5.2.9
5-Oxoproline
L-Glutamate
EC: 6.3.1.2
L-Glutamine
EC: 2.4.2.14
5-Phosphoribosylamine
EC: 3.5.1.2
EC: 3.5.1.38
EC: 6.3.5.5
L-Cysteine
Glycine
NH3
EC: 6.3.4.16
Carbamoyl-phosphate
PepA EC:3.4.11.1
PepN EC: 3.4.11.2
PepB EC:3.4.11.23
LAP3
PepD EC:3.4.13.-
DUG1
EC: 1.2.1.88
L-1-Pyrroline-5-carboxylate
EC: 6.3.2.2
L-γ-Glutamyl-L-amino acid
L-Cysteinyl glycine
EC: 2.3.2.2
EC: 3.4.19.13
EC: 1.8.1.7
GSSG
GSH
EC: 6.3.2.3
L-γ-Glutamylcysteine
NADPH
NADP+
RX
EC: 2.5.1.18
R-S-Glutathione
EC: 2.3.2.2
L-Glutamate
R-S-Cysteinyl glycine
PepN EC: 3.4.11.2
LAP3
DUG1
Glycine O
PepA EC:3.4.11.1
PepB EC:3.4.11.23
PepD EC:3.4.13.-
R-S-Cysteine
Acetyl-CoA
EC: 2.3.1.80
Mercapturic acid
EC: 1.1.1.44
EC: 1.1.1.43
EC: 1.1.1.42
EC: 1.1.1.49
EC: 1.8.4.2
EC: 1.11.1.12
EC: 1.8.3.3
EC: 1.11.1.9
EC: 1.11.1.27
EC: 3.5.1.78
TryS
EC: 2.5.1.16
Putrescine
Spermidine
EC: 4.1.1.17
L-Omithine
Glutathionyl-spermidine
L7/L12 stalk
L18
L5
L15
L18e
L21e
L30
L11
L1
L44e
L32e
L10e
L13
L15e
L6
L7Ae
L24
L4e
L37e
L29
L22
L14
L3
L2
L39e
L23
L37Ae
L31e
L24e
L19e
IF3
L35
L20
L34
Large subunit
Thx
S9
S10
S14
S7
S19
S13
S3
S2
S11
S5
S18
S6
S4
S12
S8
S15
S17
S16
S20
Small subunit
Enzyme Not dectected
Enzyme Dectected but not sigificant
Enzyme Down regulated
Ribosomal proteins Up regulated
